# Single-Cell Transcriptomics Unveils the Mechanistic Role of FOSL1 in Cutaneous Wound Healing

**DOI:** 10.3390/biomedicines13061330

**Published:** 2025-05-29

**Authors:** Jingbi Meng, Ge Zheng, Yinli Luo, Ling Ge, Zhiqing Liu, Wenhua Huang, Meitong Jin, Yanli Kong, Shanhua Xu, Zhehu Jin, Longquan Pi

**Affiliations:** 1 Department of Central Laboratory, Yanbian University Hospital, Yanji 133001, China; m.jingb@outlook.com (J.M.); zhengg1618@163.com (G.Z.);; 2 Department of Dermatology, Yanbian University Hospital, Yanji 133001, China; 3 Department of Medical Cosmetology, Yanbian University Hospital, Yanji 133001, China; 4 Department of Anesthesia, Yanbian University Hospital, Yanji 133001, China

**Keywords:** EGFR pathway, FOSL1, MAPK pathway, single-cell sequencing, wound healing

## Abstract

**Background:** The skin, a complex organ vital for protecting the body against environmental challenges, undergoes a multifaceted wound healing process involving hemostasis, inflammation, proliferation, and remodeling. The transcription factor FOSL1 has been implicated in various cellular processes crucial for wound healing, including cell cycle regulation, differentiation, and apoptosis. We hypothesize that FOSL1 is a key regulator of wound healing processes. **Objective:** The objective of this study was to investigate the role of FOSL1 in cutaneous wound healing, identify the core signaling pathways involved, and assess FOSL1′s potential as a therapeutic target. **Method:** We utilized datasets from the Gene Expression Omnibus (GEO) and applied the ‘limma’ package to discern differentially expressed genes (DEGs). We intersected these DEGs with transcription factor-associated genes from the TRRUST database. Subsequently, we constructed Protein–Protein Interaction (PPI) networks via the STRING database. Machine learning algorithms were instrumental in identifying pivotal genes, a finding corroborated through animal modeling and Western blot analysis of tissue samples. To elucidate biological pathway activities from gene expression data, we deployed the ‘PROGENy’ package, complemented by machine learning for precise pathway identification. Furthermore, Gene Set Variation Analysis (GSVA) was executed across Hallmark, biological process (BP), molecular function (MF), and cellular component (CC) categories to deepen our understanding of the wound healing process. **Results:** Our analysis revealed that FOSL1 is significantly upregulated in wounded skin. The Mitogen-Activated Protein Kinase (MAPK) and Epidermal Growth Factor Receptor (EGFR) pathways were identified as significantly associated with FOSL1. GSVA identifies critical changes in wound healing processes like ‘apical junction’ and ‘epithelial–mesenchymal transition.’ The upregulation of ‘cytoplasm organization’ and ‘response to gravity’ suggests roles in cellular adaptation. Molecular function analysis indicates alterations in ‘cytokeratin filaments’ and ‘growth factor binding,’ which are key for tissue repair. Cellular component shifts in ‘postsynaptic cytosol’ and ‘endoplasmic reticulum’ suggest changes in communication and protein processing. **Conclusions:** Our study identifies FOSL1 as a potential regulator of cutaneous wound healing through its modulation of cellular signaling pathways, offering novel insights into the molecular control of tissue repair. These findings highlight FOSL1 as a promising therapeutic target to accelerate healing in chronic or impaired wounds.

## 1. Introduction

The skin, an essential organ comprising the epidermis, dermis, and subcutaneous tissue, is the body’s first line of defense against environmental challenges. Its structural integrity is frequently compromised by injuries, from minor abrasions to severe lacerations. The wound healing process is a complex, meticulously orchestrated cascade involving hemostasis, inflammation, proliferation, and remodeling [1,2]. Each phase is characterized by the intricate interplay of cellular components, including platelets, neutrophils, macrophages, fibroblasts, and keratinocytes, all working synergistically to restore tissue integrity [2]. Hemostasis initiates the healing process, where the vascular response to injury leads to clot formation, temporarily staunching blood loss. This is swiftly followed by an inflammatory response, marked by the infiltration of neutrophils and macrophages, which not only combat potential infection but also clear debris and set the stage for the subsequent proliferative phase. During proliferation, fibroblasts and endothelial cells migrate to the wound site, laying down a new extracellular matrix and new vessels to support the growing tissue. Re-epithelialization by keratinocytes re-establishes the epithelial barrier, while collagen deposition by fibroblasts provides strength and structure to the healing tissue.

In the context of skin wound healing, the transcription factor FOSL1 has emerged as a significant player. Initially recognized for its role in embryonic development and bone matrix protein synthesis, FOSL1 has been implicated in various cellular processes, including cell cycle regulation, differentiation, and apoptosis [3]. These processes are crucial for wound healing, as they orchestrate the coordinated response of cells to injury and facilitate tissue repair. Moreover, FOSL1 has been identified as a critical regulator in the basal layer of the skin, where it modulates the expression of genes involved in the maintenance of skin integrity and the initiation of wound healing [4].

The Mitogen-Activated Protein Kinase (MAPK) signaling pathway is integral to the regulation of gene expression and cell cycle progression, which are essential for the cellular response to injury and subsequent healing [5]. Additionally, the Epidermal Growth Factor Receptor (EGFR) pathway, activated by growth factors like the Epidermal Growth Factor (EGF), is crucial for cell proliferation and migration during the proliferation phase of wound healing [6]. The MAPK pathway, through its phosphorylation cascade, can influence the activity of FOSL1, thereby affecting the transcription of genes involved in wound healing. Similarly, the EGFR pathway, upon activation, can lead to downstream signaling events that may overlap with FOSL1′s function, potentially modulating the healing process [6].

This study aims to explore the differential expression of FOSL1 in response to skin injury and its potential interactions with the MAPK and EGFR pathways. By examining the role of FOSL1 in these signaling cascades, we seek to uncover the molecular mechanisms underlying skin repair. Specifically, we hypothesize that FOSL1 is a critical regulator of wound healing processes and that modulating its activity could enhance the body’s natural healing mechanisms. The findings from this study could provide valuable insights into the molecular underpinnings of wound healing and may contribute to the development of novel therapeutic strategies that target FOSL1 to enhance the body’s natural healing processes.

## 2. Materials and Methods

### 2.1. Data Collection and Batch Correction

To analyze the gene expression associated with the cutaneous wound healing process, we curated a selection of wounded tissue-related gene expression datasets from the Gene Expression Omnibus (GEO) database (https://www.ncbi.nlm.nih.gov/geo/ (accessed on 1 March 2025)). Utilizing the search terms ‘cutaneous wound healing’, ‘cutaneous wound repair’, ‘skin wound healing’, and ‘skin wound repair’, we identified the following four datasets: GSE28914, GSE50425, GSE142471, and GSE245864 (Table 1). To mitigate the impact of batch effects on our analytical results, we applied the ‘limma’ package to correct for batch effects. For the Principal Component Analysis (PCA) and visualization, we utilized the ‘FactoMineR’ package to perform PCA on normalized data [7]. To identify transcription factor (TF)-associated genes, we leveraged the TRRUST database [8].

### 2.2. Identification of Differentially Expressed Genes (DEGs)

For the identification of DEGs, we employed the ‘limma’ package in R [9]. DEGs were determined by a threshold of log2 fold change (logFC) of ≥2 or ≤−2, signifying a substantial shift in gene expression. Additionally, we set an adjusted *p*-value threshold at 0.05, after applying the Benjamini–Hochberg method to control the false discovery rate (FDR).

### 2.3. TRRUST–STRING Integrated Gene–PPI Analysis

For the intersection of DEGs with transcriptional gene sets obtained from the TRRUST database, we employed the jvenn online tool [10]. Following the identification of these intersecting genes, we proceeded with the construction of a Protein–Protein Interaction (PPI) network using the STRING database [11]. The PPI network generated by STRING was then analyzed to identify key factors that may play central roles in the biological processes.

### 2.4. Feature Selection with Machine Learning Algorithms

For feature selection, we implemented Random Forest (RF) (ntree = 100, mtry = 10) and Support Vector Machine–Recursive Feature Elimination (SVM-RFE) (radial kernel, 5-fold cross-validation) algorithms using the R packages ‘Randomforest’ and ‘caret’ [12,13,14]. We selected features that were consistently ranked highly by both methods. This streamlined approach ensured a focused set of predictors for our models, validated through cross-validation for reliability.

### 2.5. Animal Study Design

We utilized 30 female BALB/C mice, 7 weeks old and weighing 20 g, sourced from Yanbian University Experimental Animal Center. These mice were randomly divided into five groups as follows: a normal control group on day 0 and wound model groups on days 4, 7, 10, and 14 post-wounding. This study was monitored by the Yanbian University Animal Ethics Committee (Issue No. YD20230801008). After a week of acclimatization, mice were anesthetized with 1% pentobarbital sodium, and the hair from their dorsal area was removed using depilatory cream. Full-thickness skin wounds were created using a 6 mm punch, resulting in two wounds per mouse, spaced over 1 cm apart. Mice were then group-housed and observed for health and infection. Photographs of the wounds were taken at specified time points under consistent lighting conditions. For tissue sampling, mice were anesthetized, and wound tissues were harvested, trimmed, and cleaned of excess fat and connective tissue. The samples were snap-frozen in liquid nitrogen and fixed in 4% formalin for analysis.

### 2.6. Western Blot

To assess FOSL1 protein expression during wound healing, we performed Western Blot analysis. Total protein was extracted from mouse tissue samples using RIPA lysis buffer supplemented with protease inhibitors. Protein concentration was determined using the BCA assay, and standardized samples were denatured. Proteins were separated on 8% polyacrylamide gels and transferred to PVDF membranes. Membranes were blocked with 5% non-fat milk and incubated with a primary antibody against FOSL1 (1:1000 dilution; Santa Cruz Biotechnology, Dallas, Texas, America) overnight at 4 °C, followed by a secondary antibody for 1 h at room temperature. To ensure equal protein loading and normalization, the same membrane was stripped and reprobed with a monoclonal anti-GAPDH antibody (1:10,000 dilution; Cell Signaling Technology, Danvers, Massachusetts, America) as an internal control. Protein bands were visualized using Immobilon Western chemiluminescent HRP substrate and quantified using ImageJ software (Version 1.54p).

### 2.7. Single-Cell RNA Sequencing Data Preprocessing and Pseudotime Analysis

For the preprocessing of single-cell RNA sequencing data, we utilized the Seurat package in R to normalize and identify variable features across the single-cell dataset [15]. This process included the log-normalization and scaling of expression values. To correct for batch effects and ensure consistency across samples, we employed the Harmony package, which allowed us to harmonize the data by adjusting for batch-specific variations [16]. For pseudotime analysis, we utilized the Monocle3 package [17].

### 2.8. Enrichment Analysis

We utilized the ‘PROGENy’ package to deduce the activities of biological pathways from the gene expression data [18]. To further explore the functional implications of the gene expression profiles, we used the Gene Set Variation Analysis (GSVA) package [19]. These analyses were applied to the entire dataset of gene expressions, with gene sets obtained from the ‘msigdbr’ package [20].

### 2.9. Statistical Framework and Visualization

Student’s *t*-test was selected to compare the two independent groups. We conducted these analyses using GraphPad Prism 8.0. Our programming approach in R (Version 4.4.1) included data manipulation, visualization, and statistical testing.

## 3. Results

### 3.1. FOSL1 Identification Through Comprehensive Data Analysis

**Figure 1:**
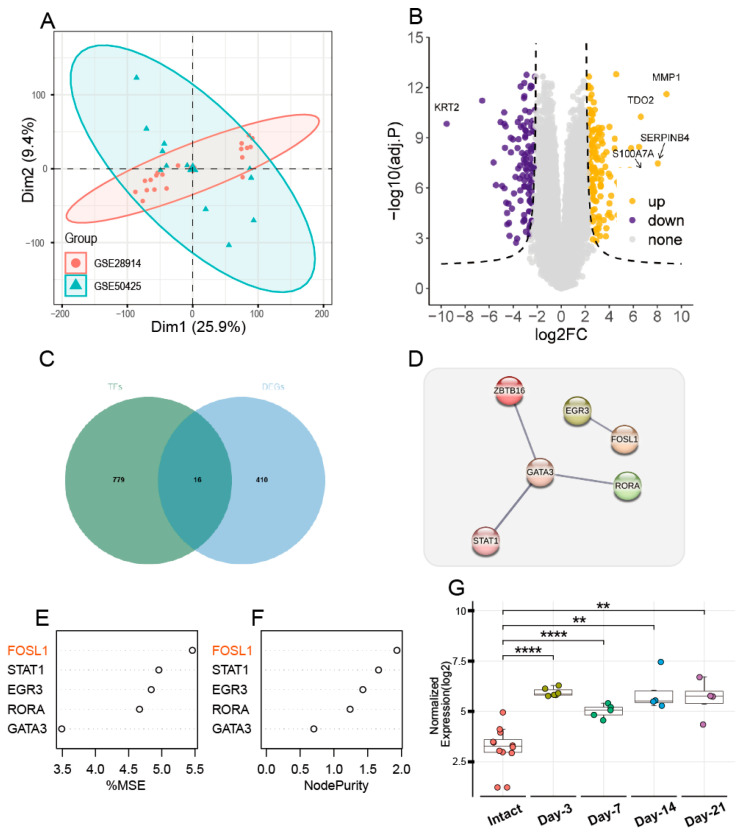
Analysis of the gene expression and network interactions in the cutaneous wound healing datasets. (**A**) PCA plot illustrating the separation of gene expression profiles between control and wound healing samples. (**B**) Volcano plot showing DEGs with logFC and negative log10 of the adj.P. (**C**) Venn diagram representing the intersection of DEGs with transcription factor-associated genes from the TRRUST database. (**D**) PPI network constructed using the STRING database, highlighting key proteins involved in wound healing. (**E**,**F**) Random Forest feature importance plot. (**G**) Box plot comparing the expression levels of FOSL1 in control and wound healing samples. ** *p* < 0.01; **** *p* < 0.0001.

After batch correction, the PCA plot confirmed a reduction in sample variance, signifying effective normalization (Figure 1A) and identifying 426 DEGs (Figure 1B, Supplementary Material S1). To explore the overlap between these DEGs and the TRRUST database’s transcription factor-associated genes, a Venn diagram analysis was conducted. It highlighted 16 core genes (Figure 1C, Supplementary Material S2). Following the Protein–Protein Interaction (PPI) analysis, we have identified the following six pivotal targets: ZBTB16, GATA3, RORA, STAT1, FOSL1, and EGR3 (Figure 1D). After feature selection using the random forest function, it was found that FOSL1 is the most important gene. After visualizing the expression of FOSL1 in the dataset, it was observed that the expression of FOSL1 after skin injury is significantly higher than that in intact skin, with the most pronounced expression observed on the third and seventh days post-injury (*p* < 0.001, Figure 1G).

### 3.2. Animal Experiments

**Figure 2:**
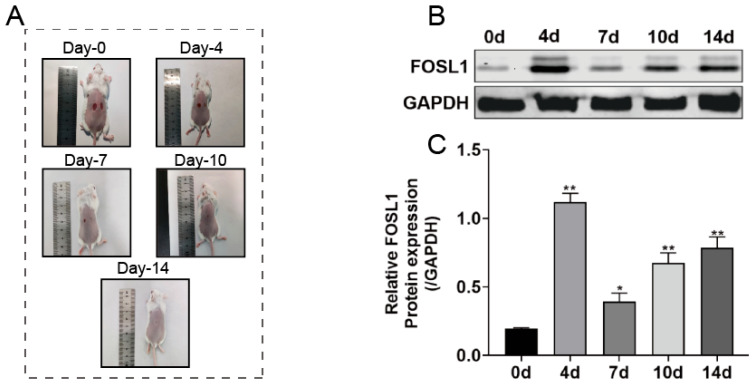
Analysis of FOSL1 expression during wound healing in an animal model. (**A**) Macroscopic images of cutaneous wound healing in the mouse model. (**B**,**C**) Western Blot analysis of FOSL1 expression. * *p* < 0.05; ** *p* < 0.01.

The results show that FOSL1 is highly expressed on days 4, 7, 10, and 14 post-wounding in wounded skin mice compared to intact skin mice, with the greatest difference in FOSL1 expression observed on the fourth day (*p* < 0.01, Figure 2A–C).

### 3.3. Identification of Cellular Composition in Wounded and Intact Samples

**Figure 3:**
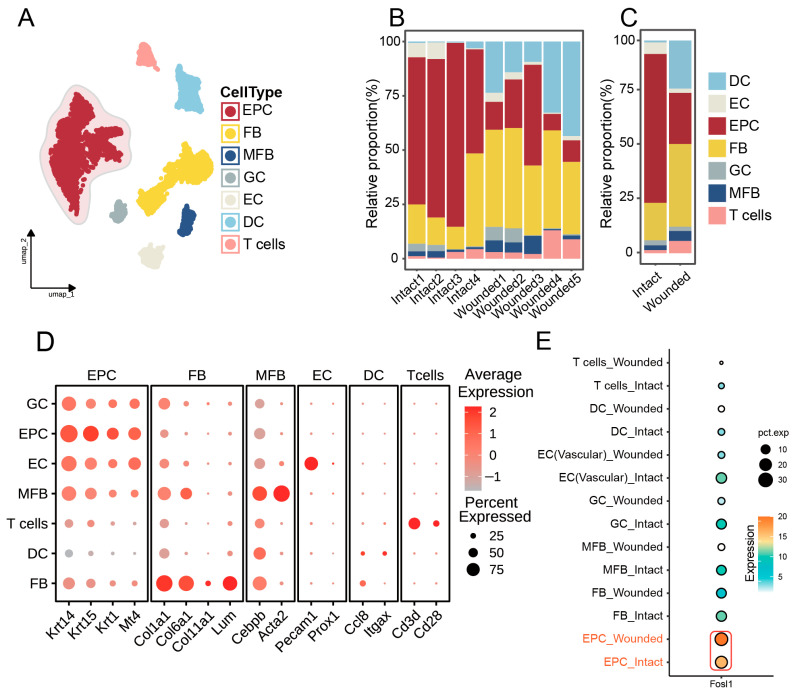
Single-cell transcriptomic analysis in the dorsal skin of wounded skin and intact skin mice. (**A**) Uniform Manifold Approximation and Projection (UMAP) clustering revealing distinct cell subpopulations. (**B**,**C**) Stacked bar charts comparing the relative abundance of each cell type between wounded and intact skin. (**D**) Dotplot showcasing the expression of key markers for each merged cell cluster. (**E**) Dotplot illustrating the expression levels of FOSL1 across various cell types, highlighting its differential expression in wounded versus intact skin. Abbreviations: epithelial cells (EPCs), T cells, dendritic cells (DCs), endothelial cells (ECs), myofibroblasts (MFBs), glial cells (GCs), and fibroblasts (FBs).

In this investigation, single-cell sequencing data from wounded skin and intact skin were retrieved from the GEO database, identified as datasets GSE142471 and GSE245864 (Table 1). Low-quality cells were excluded based on thresholds for mitochondrial gene content (>12%), unique RNA molecule counts (>5000), and total RNA counts (nCounts <500 or >15,000) (Figure S1A). ClusterTree analysis confirmed that integrated samples retained distinct biological clustering patterns without cross-platform contamination (Figure S1B). Finally, UMAP embedding visualized the corrected data, demonstrating the effective overlap of cells across platforms and time points while preserving group-specific differences (Figure S1C). After clustering analysis, we segregated cells within dorsal skin tissues into multiple subgroups, including epithelial cells (EPCs), T cells, dendritic cells (DCs), endothelial cells (ECs), myofibroblasts (MFBs), glial cells (GCs), and fibroblasts (FBs) (Figure 3A,D). Cell ratio diagrams further revealed a notably higher proportion of EPCs in intact skin than in wounded skin (Figure 3B,C). Analysis of FOSL1 expression across various cell types in both intact skin and wounded skin groups revealed a marked elevation in EPCs compared to other cellular populations (Figure 3E). Furthermore, there was a significant upregulation of FOSL1 in the wounded skin group when compared with the intact skin group, suggesting the potential role of FOSL1 in the wound healing response (Figure 3E).

### 3.4. FOSL1 Expression and Cellular Dynamics in Basal Cells During Wound Repair

**Figure 4:**
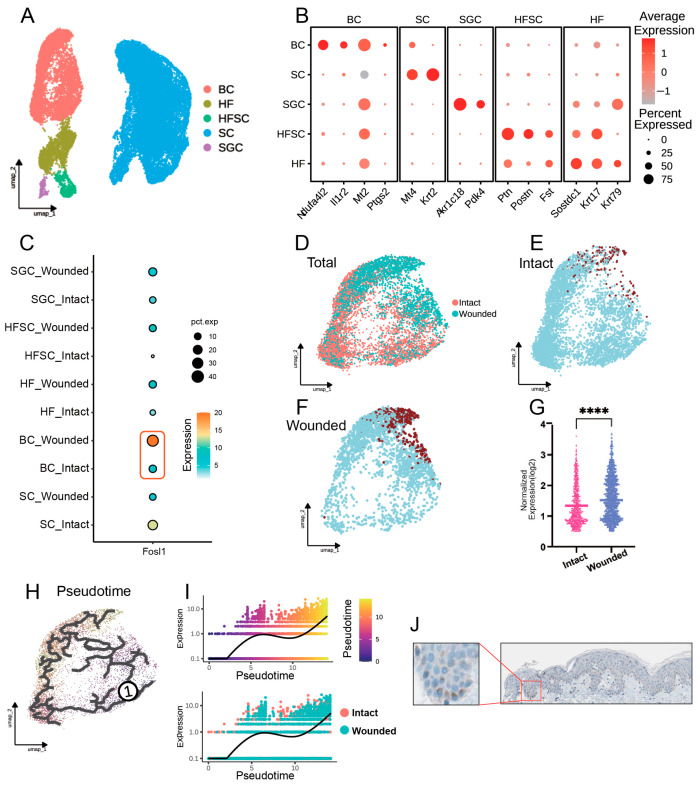
Secondary clustering and analysis of single-cell datasets. (**A**) UMAP visualization of further subdivided EPCs, showcasing the heterogeneity within this cell population. (**B**) Dotplot representing the expression of key marker genes used for cell clustering. (**C**) Dotplot illustrating the expression levels of FOSL1 across different EPC subpopulations in both intact and wounded skin groups. (**D**) UMAP plot of the basal cell population. (**E**,**F**) Comparative UMAP plots of basal cells from wounded and intact skin groups, respectively. Brown dots indicate cells expressing FOSL1. (**G**) Scatter plot depicting the differential expression of genes between intact and wounded skin, with a focus on FOSL1. (**H**) UMAP plot of cellular trajectories, with the starting point set as intact skin. (**I**) Graph showing the expression trend of FOSL1 along the cellular trajectory, emphasizing its upregulation as cells progress from intact to wounded states. (**J**) IHC images from the HPA database showing FOSL1 expression in the basal cell layer of the skin. **** *p* < 0.0001.

Following the secondary clustering of our single-cell datasets, we conducted a detailed analysis to examine the expression of FOSL1 across various cell groups in both intact and wounded skin conditions (Figure 4A,B). We also verified the expression area of FOSL1 in the skin through the Human Protein Atlas (HPA) database and found that it is significantly expressed in the basal layer (Figure 4J). Our analysis showed that the wounded basal cell group exhibited the most significant difference in FOSL1 expression (*p* < 0.001), suggesting a potential role of FOSL1 in the wound healing response (Figure 4D–G). Subsequently, we performed trajectory analysis with intact skin as the starting point (Figure 4H). The results revealed a clear upward trend in FOSL1 expression as cells transitioned from an intact to a wounded state, indicating the dynamic regulation of FOSL1 that may be integral to the wound healing process (Figure 4I).

### 3.5. FOSL1-Mediated Signaling Pathways in Basal Cells During Wound Healing

**Figure 5:**
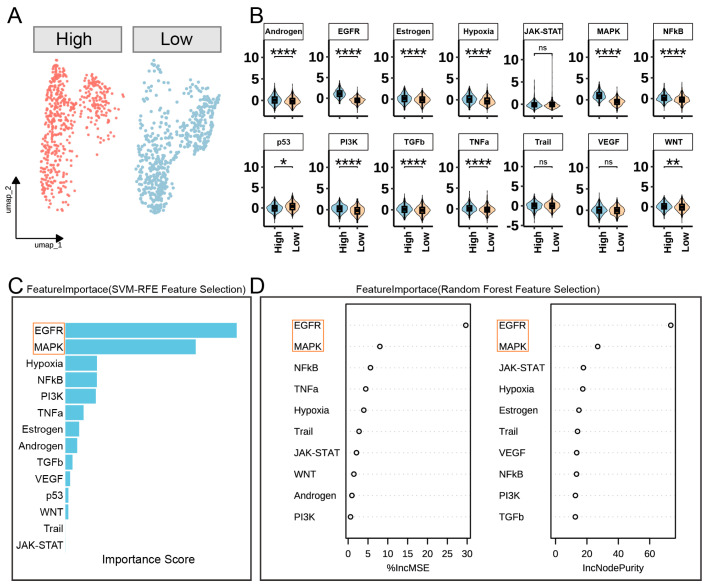
Differential signaling pathway analysis in basal cells based on FOSL1 expression levels. (**A**) UMAP plot of basal cells divided into high- and low-FOSL1 expression groups based on the median expression level. (**B**) Violin plots showing the differential activation of signaling pathways between high- and low-FOSL1 expression groups as analyzed by PROGENy. (**C**) Bar plot from SVM-RFE feature selection highlighting the importance scores of signaling pathways. (**D**) Random Forest feature selection plots showing the Percentage Increase in Mean Squared Error (%IncMSE) and Increase in Node Purity (IncNodePurity) for each pathway. * *p* < 0.05; ** *p* < 0.01; **** *p* < 0.0001; ns: no statistical significant differences.

In our analysis of basal cells stratified by FOSL1 expression levels, we utilized UMAP to visualize the cellular heterogeneity and identified two distinct groups based on median FOSL1 expression (Figure 5A). By employing the PROGENy analysis, we conducted a differential signaling pathway analysis between the high- and low-FOSL1 expression groups (Figure 5B). Our results reveal significant differences in the activation of several pathways, including the Androgen, EGFR, Estrogen, Hypoxia, MAPK, NFkB, PI3K, TGFβ, and TNFα pathways (*p* < 0.001) (Figure 5B). Interestingly, the p53 pathway was significantly downregulated in the high-FOSL1 expression group, suggesting a complex regulatory role of FOSL1 in basal cells.

To further refine our understanding of the key pathways involved, we performed feature selection using the following two complementary methods: SVM-RFE and Random Forest. The SVM-RFE feature selection (Figure 5C) identified the EGFR and MAPK pathways as the most significant, as indicated by their high importance scores. This was corroborated by the Random Forest feature selection, which used %IncMSE and IncNodePurity metrics to highlight the EGFR and MAPK pathways as the most influential in our model (Figure 5D).

### 3.6. GSVA

**Figure 6:**
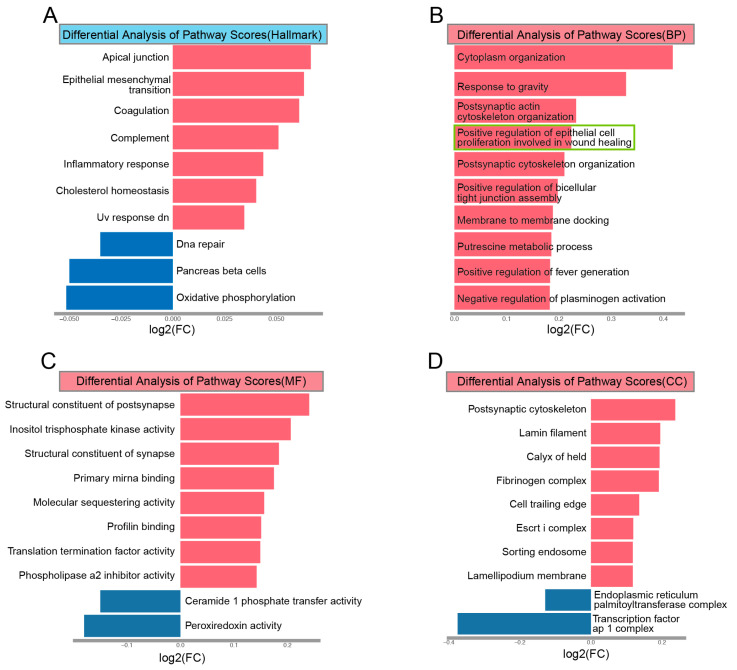
Key pathway activation in skin wound healing. (**A**) Bar plot of differential pathway scores (Hallmark), indicating significant changes in ‘apical junction’, ‘epithelial–mesenchymal transition’, and ‘oxidative phosphorylation’. (**B**) Bar plot of differential pathway scores (BP), with prominent upregulation in ‘cytoplasm organization’, ‘response to gravity’, ‘postsynaptic actin cytoskeleton organization’, and ‘positive regulation of epithelial cell proliferation involved in wound healing’. (**C**) Bar plot of differential pathway scores (MF), highlighting ‘structural constituent of polymeric cytokeratin filaments’ and ‘insulin-like growth factor binding’. (**D**) Bar plot of differential pathway scores (CC), focusing on ‘postsynaptic cytosol’ and ‘luminal surface of endoplasmic reticulum’.

In our GSVA of differential pathway scores, we identified significant alterations in various biological processes, molecular functions, and cellular components associated with skin wound healing. The Hallmark pathway analysis (Figure 6A) revealed the upregulation of ‘apical junction’ and ‘epithelial–mesenchymal transition’, alongside the downregulation of ‘oxidative phosphorylation’, suggesting a shift towards a more proliferative and migratory phenotype in wounded skin.

Our differential pathway analysis identified key biological processes (BPs) significantly upregulated in response to skin wound healing. Notably, ‘cytoplasm organization’ and ‘response to gravity’ were prominent, suggesting their role in cellular structural adaptation and orientation during the healing process (Figure 6B). Additionally, ‘postsynaptic actin cytoskeleton organization’ and ‘positive regulation of epithelial cell proliferation involved in wound healing’ were enhanced, indicating a potential contribution to cellular migration and proliferation, which are crucial for tissue repair. Molecular function (MF) analysis (Figure 6C) showed significant changes in the ‘structural constituent of polymeric cytokeratin filaments’ and ‘insulin-like growth factor binding’, pointing towards alterations in the structural integrity and growth factor signaling necessary for tissue repair. The cellular component (CC) analysis (Figure 6D) highlighted ‘postsynaptic cytosol’ and the ‘luminal surface of endoplasmic reticulum’ as key components with altered scores, which could be indicative of changes in cellular communication and protein processing during wound healing.

## 4. Discussion

This study provides an analysis of the transcription factor FOSL1 in the context of skin wound healing. Our findings underscore the multifaceted role of FOSL1 in regulating cellular processes that are pivotal to the wound healing cascade. The differential expression of FOSL1 and Western Blot analysis suggest the dynamic modulation of this factor in response to skin injury.

Our data indicate that FOSL1 is significantly upregulated in wounded skin, particularly in basal cells, which are known to be critical in the initiation and progression of the wound healing process. The strength of this study lies in the integration of multi-omics analysis, animal model validation, and single-cell transcriptomic profiling, which comprehensively dissect the mechanistic role of FOSL1 in cutaneous wound healing. By combining the bioinformatics mining of public databases with experimental validation, we not only confirmed the dynamic expression of FOSL1 but also revealed its association with the MAPK and EGFR pathways.

This upregulation aligns with the known functions of FOSL1 in promoting cell proliferation and differentiation—processes that are essential during the proliferation and remodeling phases of wound healing [21]. The observed increase in FOSL1 expression during these phases suggests that FOSL1 may act as a positive regulator of tissue repair.

A significant finding of our study is the association of FOSL1 with both the MAPK and EGFR signaling pathways, which are pivotal in mediating growth factor responses and orchestrating cellular activities essential for wound healing. Our findings imply that FOSL1 could act as a potential connector, integrating the signaling cascades initiated by these pathways to modulate the transcriptional regulation of genes that play a role in tissue repair.

The inhibition of the MAPK pathway has been shown to result in delayed wound closure, underscoring its significance in the healing process [22]. The EGFR pathway also plays a pivotal role in cutaneous wound healing, particularly in the regulation of basal cells [23]. EGFR deficiency significantly delays wound closure, indicating it is a critical function in the healing process [23]. The absence of EGFR leads to increased edema, prolonged eschar formation, and impaired keratinocyte migration and proliferation. Notably, the influence of the EGFR signaling pathway on basal cells is a key determinant in the wound healing cascade, suggesting that the modulation of EGFR signaling could be a therapeutic strategy to enhance healing outcomes. Furthermore, it is imperative to consider the implications of EGFR inhibition, which is commonly used in cancer therapy and can induce dermatological toxicities such as papulopustular rashes [24]. The prolonged use of these inhibitors may also suppress wound healing [25]. This highlights the need for a nuanced understanding of EGFR signaling in different contexts and the development of therapeutic strategies that can modulate its activity to promote healing without compromising cancer treatment outcomes.

Our study’s findings are consistent with those of previous research that implicates FOSL1 in various cellular processes relevant to wound healing. However, our study extends these findings by providing an analysis of FOSL1′s expression and its potential regulatory role in the context of skin injury.

Our study has several limitations. First, direct mechanistic evidence (e.g., ChIP-seq or functional assays) is lacking to confirm FOSL1′s transcriptional regulation of MAPK/EGFR effectors. Second, the exclusive use of female mice limits generalizability to sex-specific responses in wound healing. Third, while single-cell analysis revealed FOSL1 dynamics in basal cells, cellular-level functional validation is needed to define its precise role. Fourth, the absence of exogenous FOSL1 modulation hinders potential clinical assessment. Finally, the GSVA-derived pathway associations require experimental confirmation. Future studies should address these gaps by incorporating male animal models, human clinical samples, and mechanistic validations to strengthen translational relevance.

Collectively, our findings position FOSL1 as a potential therapeutic target. The observed upregulation of FOSL1 in basal cells and its crosstalk with the EGFR and MAPK pathways suggest that the targeted modulation of FOSL1 activity—through localized agonist delivery or combinatorial approaches with existing EGFR inhibitors—may enhance the healing of chronic wounds.

## 5. Conclusions

Our study provides novel insights into the role of FOSL1 in skin wound healing. The study’s findings suggest that FOSL1 could be a critical regulator of the process, with potential implications for the development of therapeutic strategies. By targeting FOSL1, it may be possible to enhance the body’s natural healing mechanisms and improve outcomes for patients with skin injuries.

## Figures and Tables

**Table 1:** Summary of external datasets used for analyses.

Accession	Country	Sample	Organism	Wound Date	Data Type	Manufacturer	Platform
GSE28914	Finland	Tissue	Homo sapiens	Intact, 3rd, 7th	Chip	Affymetrix	U133 Plus 2.0 Array
GSE50425	Finland	Tissue	Homo sapiens	Intact, 14th, 21st	Chip	Illumina	HumanHT-12
GSE142471	USA	Tissue	Mus musculus	Intact, 4th	Single cell	Illumina	Hiseq 4000
GSE245864	USA	Tissue	Mus musculus	Intact, 3rd	Single cell	MGItech	DNBSEQ-G400

## Data Availability

The datasets generated during this study are available in open access repositories. The custom code used for data analysis is accessible at https://github.com/md26meng/Fosl1 (accessed on 1 March 2025). All other data supporting the findings of this study are available from the corresponding author upon reasonable request.

## Supplementary Materials

The following supporting information can be downloaded at: https://www.mdpi.com/article/10.3390/biomedicines13061330/s1, Figure S1: Preprocessing; Supplementary Material S1: total DEGs; Supplementary Material S2: Intersected results.
