# Supplementary figures and images for: Single-Cell Transcriptomics Unveils the Mechanistic Role of FOSL1 in Cutaneous Wound Healing
Source: Biomedicines. 2025 May 29;13(6):1330. doi: 10.3390/biomedicines13061330 (PMC12189721; doi:10.3390/biomedicines13061330)

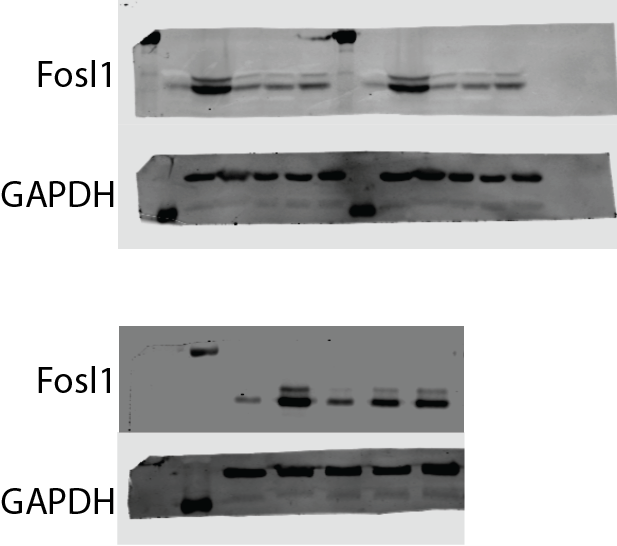

Supplement: Supplementary file 1 [file biomedicines-13-01330-s001.zip › Supplement Figures/WB_raw_bands.png]

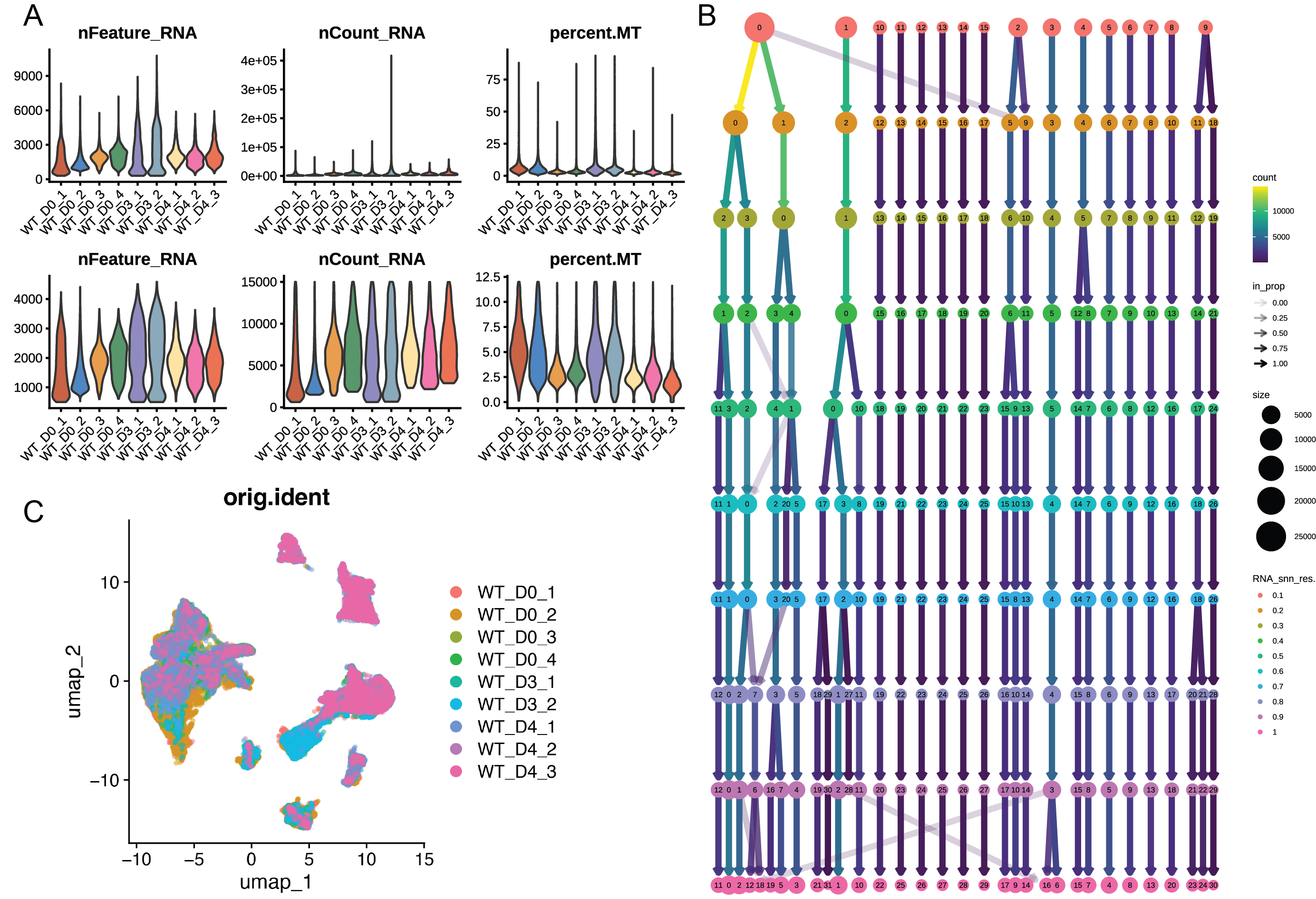

Supplement: Supplementary file 1 [file biomedicines-13-01330-s001.zip › Supplement Figures/Figure S1.png]
